# Distinct Cis Regulatory Elements Govern the Expression of TAG1 in Embryonic Sensory Ganglia and Spinal Cord

**DOI:** 10.1371/journal.pone.0057960

**Published:** 2013-02-26

**Authors:** Yoav Hadas, Noa Nitzan, Andrew J. W. Furley, Serguei V. Kozlov, Avihu Klar

**Affiliations:** 1 Dept. of medical neurobiology, IMRIC, Hebrew University-Hadassah Medical School, Jerusalem, Israel; 2 Department of Biomedical Science, University of Sheffield, Western Bank, Sheffield, United Kingdom; 3 Center for Advanced Preclinical Research, SAIC-Frederick, Inc., Frederick National Laboratory for Cancer Research (FNLCR), Frederick, Maryland, United States of America; Institut de la Vision, France

## Abstract

Cell fate commitment of spinal progenitor neurons is initiated by long-range, midline-derived, morphogens that regulate an array of transcription factors that, in turn, act sequentially or in parallel to control neuronal differentiation. Included among these are transcription factors that regulate the expression of receptors for guidance cues, thereby determining axonal trajectories. The Ig/FNIII superfamily molecules TAG1/Axonin1/CNTN2 (TAG1) and Neurofascin (Nfasc) are co-expressed in numerous neuronal cell types in the CNS and PNS – for example motor, DRG and interneurons - both promote neurite outgrowth and both are required for the architecture and function of nodes of Ranvier. The genes encoding *TAG1* and *Nfasc* are adjacent in the genome, an arrangement which is evolutionarily conserved. To study the transcriptional network that governs TAG1 and Nfasc expression in spinal motor and commissural neurons, we set out to identify cis elements that regulate their expression. Two evolutionarily conserved DNA modules, one located between the *Nfasc* and *TAG1* genes and the second directly 5′ to the first exon and encompassing the first intron of *TAG1*, were identified that direct complementary expression to the CNS and PNS, respectively, of the embryonic hindbrain and spinal cord. Sequential deletions and point mutations of the CNS enhancer element revealed a 130bp element containing three conserved E-boxes required for motor neuron expression. In combination, these two elements appear to recapitulate a major part of the pattern of TAG1 expression in the embryonic nervous system.

## Introduction

Neurons extend their axons toward intermediate and final targets with remarkable precision. The neuronal fate of neurons and their subsequent axonal trajectory are governed by transcription factors (TFs) [1], [2]. Gene networks that specify neuronal wiring in the vertebrate CNS are being actively unraveled, notably in spinal motor and interneurons [3], [4], [5]. In the spinal cord, classical surgical manipulations of the early embryo demonstrated that motor neurons are programmed to innervate their corresponding muscular target as they differentiate [6]. In the lumbar and brachial motor neurons, the choice to innervate the dorsal vs. the ventral limb muscles is encoded by an Lim-HD code: Lhx1 and Isl1 are expressed in LMCl and LMCm neurons, respectively, and are required to direct ventral versus dorsal limb innervation. This target selection by LMC neurons is manifest by the downstream activation of Eph receptors: EphA4 by Lhx1 and EphB receptors by Isl1 [3], [4], [7]. However, it is not known whether either Lhx1 or Isl1 activates Eph receptor expression directly. Subsequent innervation of specific muscles is similarly governed by the Hox code [8], but the downstream targets of the Hox TFs, presumably genes encoding receptors that decode the muscle-derived guidance cues, remain thus far elusive.

The choice of spinal interneurons, whether to project axons ipsilaterally or contralaterally, is regulated on a cellular level at several choice points. Ipsilaterally projecting neurons turn longitudinally at several dorso-ventral levels: dI3d in the dorsal funiculus (DF), dI1i in the lateral funiculus (LF), dI3v in the ventral lateral funiculus (VLF) and V2 in the ventral funiculus (VF) [9], [10], [11]. The axons of commissural interneurons are guided towards the floor plate (FP) by FP-derived attractants: Netrin, Shh and VEGF, and are desensitized to floor plate-derived repulsive molecules (Slits) via the expression of Robo3 [12]. In vitro perturbation and loss of function experiments in the chick embryo support a role for the interactions between axonal TAG1/Axonin1 and midline-expressed NrCAM in regulating the entry into the floor plate [13], [14]. Slits and their axonal receptors, Robo1 and Robo2, regulate the exit from the floor plate [15], [16].

The transcriptional programs that direct the ipsi- versus contra-lateral axonal projection are starting to unravel. Ipsilateral projection of retinal ganglion cells requires the expression of the zinc finger transcription factor Zic2 [17]. In the spinal cord the ipsilateral projection of dI1i spinal interneurons requires the expression of BarhL2 [18], and Isl1 is implicated in the ipsilateral turn of dI3v axons [19]. For the commissural projection, the Lim-HD transcription factors Lhx2 and Lhx9 were demonstrated to be required for floor plate crossing of dI1c axons [5].

How do TFs regulate the responsiveness to midline cues? Ipsilaterally projecting retinal ganglion cells are repelled by optic chiasm-derived EphrinB and Zic2 positively regulates the expression of the EphB1 receptor [17]. In dI1c neurons, Lhx2 and Lhx9 are required for the expression of Robo3, which in turn is required to suppress activation of Robo1 and Robo2 by Slit, which otherwise repel these axons from midline crossing [5]. The regulatory network that instructs commissural projection of dI1c axons is probably mediated by direct activation since binding sites for Lhx2 and Lhx9 are located in the presumed regulatory elements of Robo1, 2, and 3 [5], [20]. The ipsilateral projection of dI1i axons requires BarHL2 to downregulate Robo3 expression and in its absence dI1i axons ectopically express Robo3 and aberrantly cross the midline [18].

In the present study we have begun to elucidate the genetic networks that control the expression of TAG1 in the embryonic spinal cord and hindbrain levels. We have identified two evolutionarily conserved DNA enhancer elements, one located between the *Nfasc* and *TAG1* genes and the second directly 5′ to the first exon and encompassing the first intron of *TAG1*. These elements directed complementary expression in the CNS and PNS, respectively, of the embryonic hindbrain and spinal cord. The CNS enhancer was further characterized utilizing genome alignment and sequential deletion. An evolutionarily conserved minimal, 130 bp DNA element upstream to the *TAG1* gene, that is sufficient to drive expression in motor and subset of commissural neurons was identified. Mutagenesis analysis of putative binding sites for bHLH and Lim-HD TFs, revealed that bHLH binding sites – 3 E-boxes harbored by the 130 bp element – are required for its expression in motor neurons.

## Results

### Sequences immediately upstream of the *TAG1* gene harbor dorsal root and cranial ganglia-specific control elements of TAG1 expression

TAG1/Axonin1/CNTN2 (here referred to as TAG1) is expressed in neurons of the central and peripheral nervous system [13], [21], [22]. During early embryonic development of rodents and aves, TAG1 is expressed in commissural neurons, motor neurons, cranial ganglia and DRG neurons [13], [22], [23], [24]. However, the temporal profile of TAG1 expression in the spinal cord versus DRG neurons, both *in vivo* and *in vitro*, is dissimilar, suggesting differential regulation by central and peripheral neurons [25]. To identify the regulatory elements and decode the gene networks that govern TAG1 expression in the CNS and PNS, we aimed to identify the cis elements that control the spatial expression of TAG1 in the embryonic nervous system.

**Figure 1:**
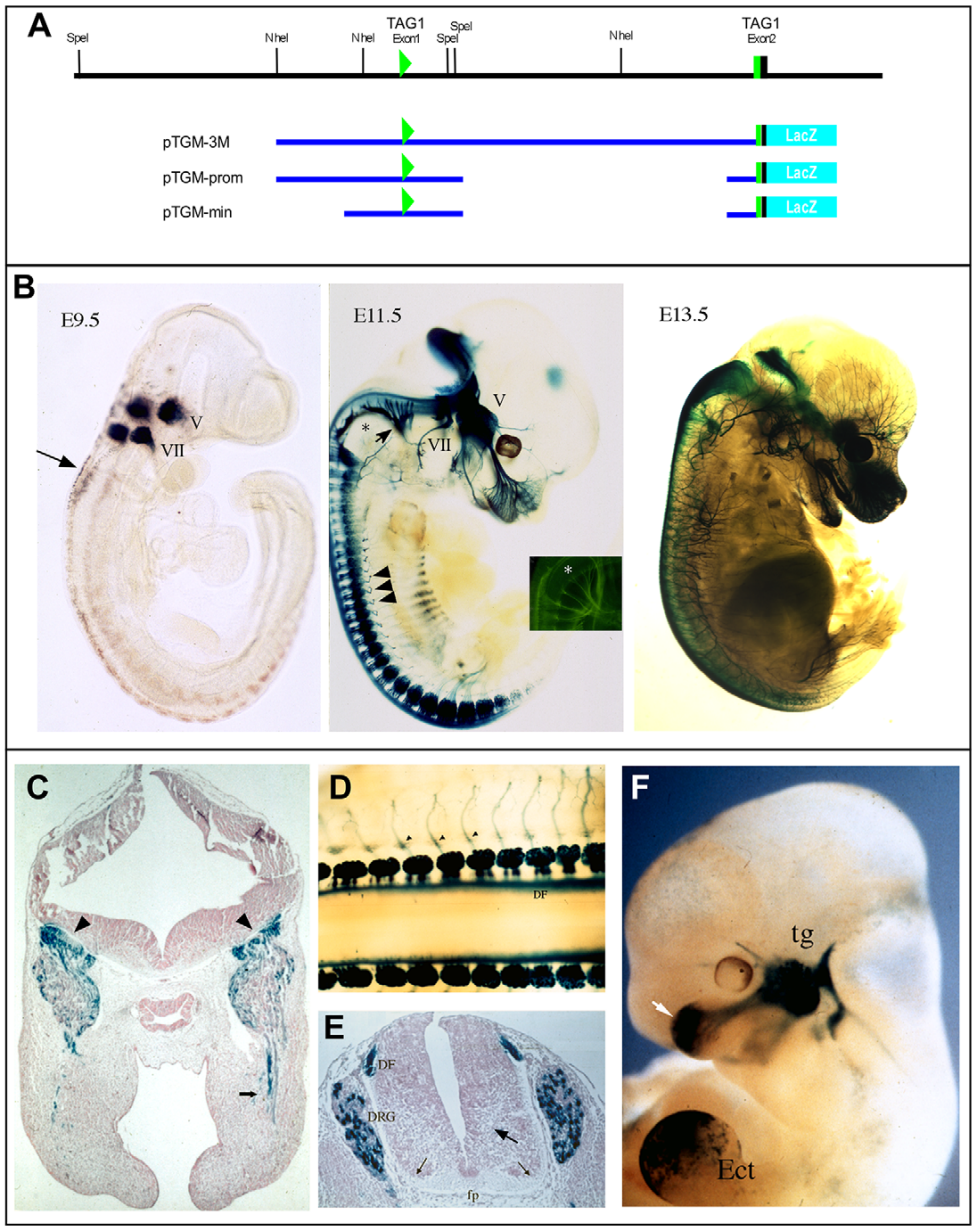
Proximal TAG1 enhancer drives expression in embryonic peripheral nervous system. A. Scheme showing organization of mouse *TAG1* gene 7 kb upstream and 8.5 kb downstream of exon 1, which is non-coding (green triangle). Includes exon2, which encodes the start methionine and leader sequence (green and black box) and exons 3–5 (black boxes) which are separated from exon 2 by a 4 kb intron, only part of which is shown. Below are indicated constructs used in this study, which include human *TAG1* genomic DNA (blue line) fused in-frame at the ATG codon to a LacZ reporter gene (light blue). B. Expression of pTGM-3M in line TAG/LacZii [26] at ages indicated, as detected by X-gal staining. Expression is first evident at E9.5 in the 5^th^ (V) and 7^th^ (VII) cranial ganglia and rostral migrating neural crest (arrow). In addition, at E11.5 staining can be seen in the ganglia of several cranial nerves, including the 10^th^ (arrow), and in spinal dorsal root ganglia (arrowheads), but not in the 12^th^ nerve (black asterisk; inset shows staining with anti-TAG1 (green) at an equivalent stage in wild type embryo, white asterisk marks 12^th^ nerve in ventral neural tube). By E13.5, most sensory subsets are seen to express the transgene. C. Section of E11.5 TAG/LacZii embryo confirms X-gal staining in CNS is due to afferents entering hindbrain from trigeminal (arrowheads) and that there are no cells expressing the transgene within the hindbrain neuroepithelium. X-gal staining is also seen in axons extending to jaw (arrow). D,E. Similarly, X-gal staining in spinal cord is due to axons entering the dorsal funiculus (DF) from the DRG and no staining can be seen in the spinal neuroepithelium, either in a dorsal view of a wholemount stained E11.5 embryo (D), or in a transverse section at the same age (E). Note particularly the absence of staining in the ventral root (small arrows), in the commissural axon pathway (large arrow) or under the floor plate (fp). Arrowheads in D indicate peripheral branches of sensory axons. F. A transient transgenic embryo at E11.5 carrying the pTGM-prom construct. Expression overall is weaker, though consistent with that of pTGM-3M transgenics - showing for example expression in the trigeminal (tg) - but complicated by frequent, inconsistent ectopic expression, present in this example in the limb (ec). White arrow shows X-gal staining in axons of trigeminal.

An ∼15 kb genomic fragment extending from ∼4 kb upstream of exon 1 (non-coding) through to the ATG start site in exon 2 of human *TAG1,* has been shown to drive expression of a LacZ reporter gene (fused in-frame immediately downstream of the ATG start site; Fig. 1A) in the postnatal cerebellum of transgenic mice in a manner similar to endogenous TAG1 [26], but its embryonic expression was not reported. Denaxa et al., 2003 [27] reported that a 4kb fragment of human genomic DNA, apparently lying directly upstream of exon 1, is sufficient to largely recapitulate the TAG1 expression pattern, including expression in the embryonic spinal cord, when placed next to a LacZ reporter gene. However, the reporter gene expression driven by this fragment was considerably weaker and more variable than endogenous TAG1 expression, and expression in motor neurons and dorsal root ganglia (DRG), among other tissues, was not seen, leading the authors to conclude that a number of regulatory elements were missing.

Therefore, since the transgenic construct (pTGM-3M; Fig. 1A) used by Bizzoca et al (2003) utilizes both the natural transcription and translation start sites of TAG1, and encompasses a substantial proportion of sequences upstream to the transcription start site and all of intron 1, which is known in the genes of other members of TAG1 family to contain critical regulatory elements [28], [29], we examined its expression in the embryonic spinal cord of our established transgenic line [26]. Strong reporter gene expression was detected as early as E9.5 in the 5^th^ (trigeminal) and 7^th^ (facial) cranial ganglia (Fig. 1B). By E11.5 expression was also evident in the ganglia of several additional cranial nerves, including the vagus (10^th^; arrow) – but not in the hypoglossal (12^th^; asterisk) or oculomotor nerves. Beta-galactosidase (ß-gal) from reporter expression was also evident in the axons of the trigeminal ganglia, including the mandibular, maxillary and ocular branches, and also in its projections into the hindbrain (Fig. 1B, C). More caudally, reporter expression was seen in neurons in the dorsal root ganglia and ß-gal was present in both their peripheral and centrally projecting axons (Fig. 1B, D, E). However, there was no evidence of reporter gene expression in neurons within the spinal cord, the only labelling within the CNS being of axons extending in from the periphery (Fig. 1D, E). Similar staining was found at the level of the trigeminal ganglia, with only incoming peripheral axons labelling within the hindbrain itself (Fig. 1C arrowheads). Analysis of a second permanent line and of transient transgenic embryos also made with pTGM-3M, indicated that this expression pattern was typical.

A similar pattern was obtained with a reporter construct lacking most of intron 1 (pTGM-prom), but expression was consistently weaker, sometimes labelling only the trigeminal, and ectopic expression was often found (Fig. 1F). A construct including just 1.5 kb upstream of exon 1 (pTGM-min), although partly recapitulating the overall pTGM-3M pattern, consistently revealed weaker and frequently ectopic expression (not shown). Notably, all the three constructs contain the 5′ region of the 1^st^ intron, that is conserved between mammals and the marsupial – opossum (Fig. S1). However, with none of the three constructs studied was there evidence of expression by cells located within the spinal cord, suggesting that the element(s) controlling commissural and motor neuron transcription of TAG1 are positioned elsewhere in the genome

### TAG-1 and Nfasc genes are closely linked and overlap in their embryonic spinal cord expression

**Figure 2:**
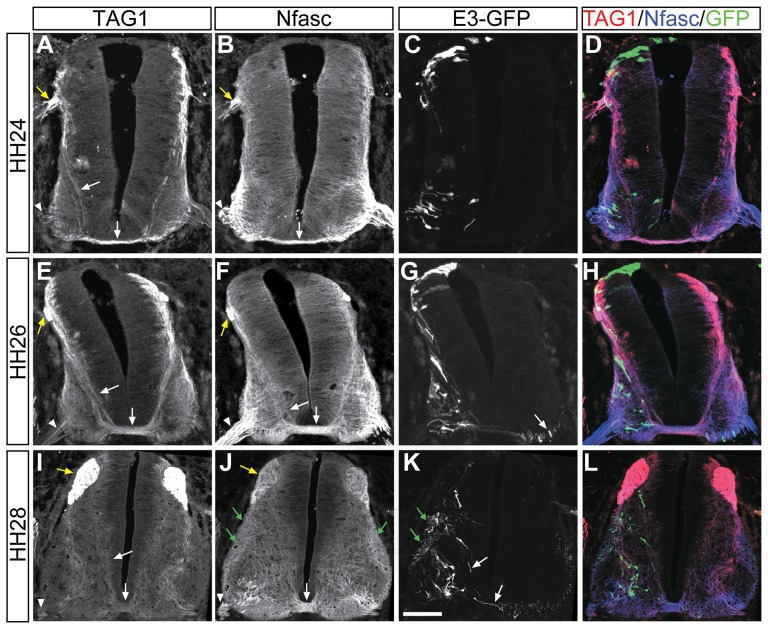
Expression pattern of TAG1, Nfasc and the E3 enhancer in the chick embryonic spinal cord. The neural tube of HH st. 18 chick embryos were electroporated with E3::Cre + CAG-STOP^LoxP^-GFP plasmids. Cross sections of HH24 (A–D), HH26 (E–H) and HH28 ( I–L) were stained for TAG1 (A,E,I), Nfasc (B,F,J) and GFP (C,G,K). The merged triple staining is shown in D,H,L. White arrows point to commissural axons. White arrowheads point to motor axons. Yellow arrows point to DRG axons. Green arrows point to ipsilaterally projecting axons. Scale Bar A–D 70 µm, E-H 90 µm, I-L 140 µm.

To identify additional potential TAG1 cis regulatory elements, we focused on sequences located between the TGM-3M region and the 3′ end of the gene lying centromeric to *TAG1*, *Neurofascin* (*Nfasc*). Neurofascin is a transmembrane-linked adhesion molecule closely related to TAG1 in structure [30]. The expression pattern in the spinal cord of both molecules is similar [31], However the relative expression of Nfasc and TAG1 has not been reported. Because of the proximity of the two genes (the distance between the 3′ end of *Nfasc* and the 5′ of *TAG1* on mouse and human chromosomes 1 is 25 kb and 20 kb, respectively, and just 2.7 kb on chick chromosome 26), we speculated that they may share regulatory elements. Consistent with this, there is substantial overlap between the two proteins in the early chick spinal cord (Fig. 2). At HH24 (Hamburger and Hamilton stages), TAG1 is expressed in DRG axons, and in subsets of motor axons and interneurons. Expression in commissural spinal interneurons is evident in axons that project diagonally toward the floor plate and in the axons that elongate at the midline (arrows, Fig. 2A). Nfasc is expressed in the same neurons, although its expression in motor and interneurons and their axons is more widespread (Fig. 2B). Interestingly, TAG1 expression is observed on pre-crossing commissural axons, while Nfasc expression is predominant on commissural axons that are crossing the floor plate (Fig. 2A,B), suggesting that the translation or the membrane localization of the proteins is subjected to different modes of regulation. Similar expression patterns are maintained at HH26 and HH28, where TAG1 is clearly expressed in a subpopulation of Nfasc expressing neurons (Fig. 2E,F,I,J). Notably, TAG1 expression is restricted to commissural axons while Nfasc is expressed also in ipsilaterally projecting axons (Fig. 2I,J). These observations are therefore consistent with the possibility that a shared regulatory element lies in the *Nfasc-TAG1* intergenic region.

### An evolutionarily conserved element between *TAG1* and *Nfasc* directs expression to motor and commissural neurons

**Figure 3:**
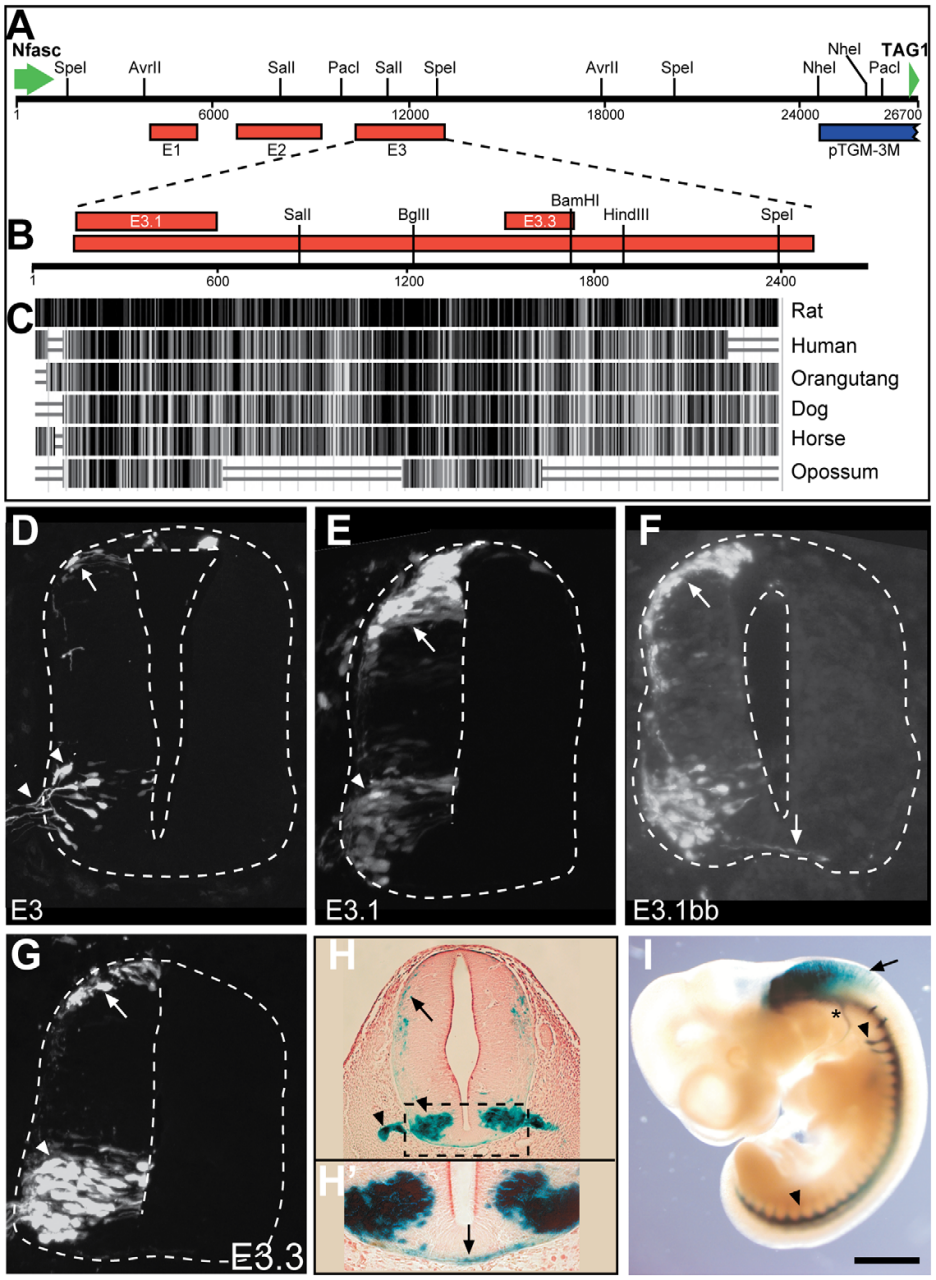
Functional dissection of the E3 enhancer element. A. A scheme showing the mouse genomic organization of the region between the 3′ of *Nfasc* and 5′ of *TAG1*. B. A zoom-in of the E3 element showing the E3.1 and E3.3 elements. C. Alignment of the mouse E3 element to the human, orangutan, dog, horse and Opossum elements. The Alignment was done using BLAT alignment tool of UCSC genome bioinformatics (http://genome.ucsc.edu/cgi-bin/hgGateway). D–G. The expression patterns of GFP driven by the indicated enhancer (at the bottom right side of each image) at HH26 chick spinal cord. Each enhancer was cloned upstream to Cre recombinase and electroporated at HH18 into the chick neural tube along with CAG-STOP^LoxP^-GFP plasmid. H,I. The expression pattern of E3.1::LacZ in E11.5 mouse embryo. The boxed area in H is shown as a magnification in H’. Arrows point to dorsal neurons and commissural axons. Arrowheads point to motor neurons and motor axons. Scale Bar A–G 50 µm, H-75 µm, H'-25 µm.

Comparative genomics and complementary transgenic approaches are demonstrated to be a reliable method for identifying enhancer elements in various tissues [32], [33], [34] including embryonic spinal cord neurons [35], [36]. Alignment of the genomic region between *TAG1* and *Nfasc*, in various mammals, utilizing UCSC alignment tools, revealed three conserved regions designated E1, E2 and E3 (Fig. 3A). Each potential enhancer element was isolated from the mouse genome and cloned upstream of Cre recombinase. These Cre plasmids were electroporated into the chick neural tube along with a Cre-dependent GFP plasmid, using the experimental paradigm used previously to identify neuronal specific enhancer elements [9], [10], [19], [36]. In contrast to the E1 and E2 elements, which yielded no expression of the reporter gene, E3 directed expression of GFP specifically in motor neurons, dorsal interneurons and roof plate cells (Fig. 2C,D,G,H,K,L, Fig 3D, table 1).

**Table 1:** Quantification of GFP labeled neurons.

Enhancer	#GFP cells	%IN	%RP	%MN
E3	1321	37.2	17.4	45.4
E.3.1	3334	48.7	4.9	46.4
E3.3	2738	41.7	5.1	53.2
E3.1Em4	1168	80.1	10.7	9.2
cE3	1150	18.7	15.3	66

Cross sections of HH24 electroporated embryos were stained with cell fate markers. Interneurons (IN) were stained with Lhx2/9, Lhx1, Isl1, Brn3a and motor neurons (MN) with Isl1. Progenitor cells that reside in the ventricular zone, that do not express any of the cell fate markers, were classified according to their dorsoventral location. Roof plate cells (RF) were defined as dorsal midline cells that do not express the dI1-3 cell fate marker Brn3a.

To further dissect the E3 enhancer element, we broadened our homology search: The homology of E3 between mammals is high along its entire 2.7 Kb. However, alignment with the genome of the marsupial Opossum, revealed two short regions of homology: E3.1 (450 bp) and E3.3 (380 bp) (Fig. 3B,C). Both E3.1 and E3.3 directed expression of GFP in motor and the dorsal interneurons, in a similar pattern to the E3 enhancer element (Fig. 3E,G, table 1). A marked difference between the long E3 and the short E3.1 and E3.3 elements is the labeling of progenitor motor neurons, residing at the ventricular zone and the medial ventral spinal cord, in the short enhancers (Fig. 3E,G), while E3 labeled the differentiated, laterally positioned motor neurons (Fig. 2K,L, Fig. 3D). Accordingly, E3-labeled motor neurons co-express TAG1 (Fig. 1G, S3), while pMN labeled with E3.1 and E3.3 have not initiated the expression of TAG1, but do express Isl1 (Fig. S3). Hence, it is likely that E3 cis elements that inhibit expression in progenitor neurons are absent in E3.1 and E3.3.

The fidelity and specificity of E3.1 enhancer was also assessed in the mouse. The enhancer was cloned upstream of the Hsp68 minimal promoter driving the expression of LacZ reporter gene. At E11.5, 9 out of 11 (82%) embryos transgenic for E3.1-Hsp68-LacZ construct revealed expression of LacZ marker restricted to spinal motor neurons, hindbrain and cervical dorsal commissural neurons (Fig. 3H,I). In contrast to the TGM constructs (Fig. 1), the E3.1 enhancer did not label the DRG or any of the cranial nerves, with the exception of the hypoglossal (Fig. 3I; asterisk). Hence, the 450 bp of the E3.1 element harbors an enhancer activity capable of driving expression in motor and commissural neurons, complementary to that seen with the TGM elements.

### E3 directs expression predominately in dI1 neurons

By HH28, coincident with the expression of Nfasc (Fig. 2J), E3-directed GFP expression (Fig. 2K, L) is found in both commissural (Fig. 2K, white arrows) and ipsilateral axons (Fig. 2K, green arrows), consistent with the idea that E3 is a 3′ enhancer of Nfasc. However, an alternative possibility is that the E3 enhancer is active in a progenitor that is common to both contra and ipsi- projecting neurons, but is then silenced in cells that become ipsi-projecting, as is characteristic of dl1 progenitors [5]. In this case, because GFP reporter expression would be permanently activated by Cre-mediated recombination in the progenitor, it would remain on in both daughter lineages. To distinguish between these two models we set out to compare the subtype of interneurons that express E3 and their axonal projection pattern.

**Figure 4:**
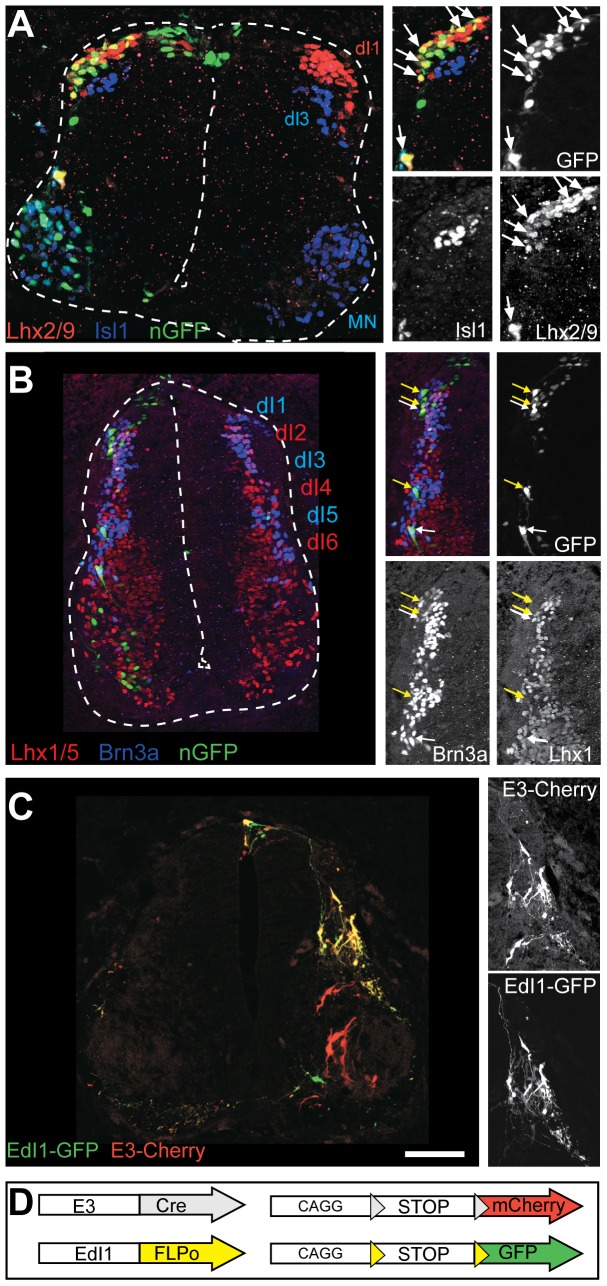
E3 enhancer directs expression in dI1 neurons. E3::Cre and CAG-STOP^LoxP^-nGFP were co-electroporated. Cross sections of HH26 spinal cord were stained with cell fate markers. In the dorsal spinal cord, most of the nGFP cells express the dI1 cell fate marker Lhx2/9 (A) and Brn3a (yellow arrows in B). Few cells express the dI2 markers Brn3a+Lhx1 (white arrows in B). dI3 (Isl1+) and dI4-6 (Lhx1+/Brn3a-) do not express nGFP (A,B). Co expression of GFP driven by dI1 specific enhancer [10], and mCherry driven by E3 enhancer (D), demonstrate overlapping expression in the dorsal interneurons (C). Scale Bar A-B 50 µm, C100 µm.

To determine whether the E3 enhancer is able to activate expression in dorsal interneurons with dl1 characteristics, GFP with a nuclear localization signal (nGFP) was electroporated into embryos under the control of E3 and cross sections were then examined with markers that identify different cell fates. Among the post mitotic neurons, 83% of the dorsal interneurons expressing the nGFP are dI1 (as defined by expression of Lhx2/9; 281 Lhx2/9^+^ neurons from 337 nGFP neurons. Neurons were counted from 5 HH26 embryos, 5 cross sections from each embryo), 9% are dI2 (31- Lhx1+/Brn3a+ neurons from 331 nGFP neurons), and 8% of the neurons were distributed between other interneurons (Fig. 4A,B). The preferential expression in dI1 neurons was tested by comparing the soma and axonal labeling of the E3 enhancer element with that of the dI1 specific enhancer element – EdI1. EdI1 directs reporter expression in the two dI1-derived subpopulation: dI1c and dI1i [10]. E3::Cre and EdI1::FLPo were simultaneously electroporated along with two corresponding reporter cassettes: Cre-dependent mCherry and FLP-dependent GFP, respectively (Fig. 4D). All the mCherry expressing dorsal interneurons co expressed GFP, while motor neurons expressed only mCherry (Fig. 4C). GFP+/mCherry- neurons are settled at the ventral lateral spinal cord (Fig. S2), position that is populated with the ipsi lateral dI1 subpopulation – dI1i [5], [10]. Collectively these data provide additional evidence that E3 directs expression in the dorsal spinal cord primarily to dI1c neurons. Concomitantly, commissural axons co-expressing GFP and mCherry, and motor axons expressing only mCherry are evidenced (Fig. S2). Interestingly, many of the ipsilaterally axons are GFP+/mCherry- (Fig. S2), proving further support to the commissural expression of the E3 enhancer element.

### E3 is a potential target of bHLH transcription factors

**Figure 5:**
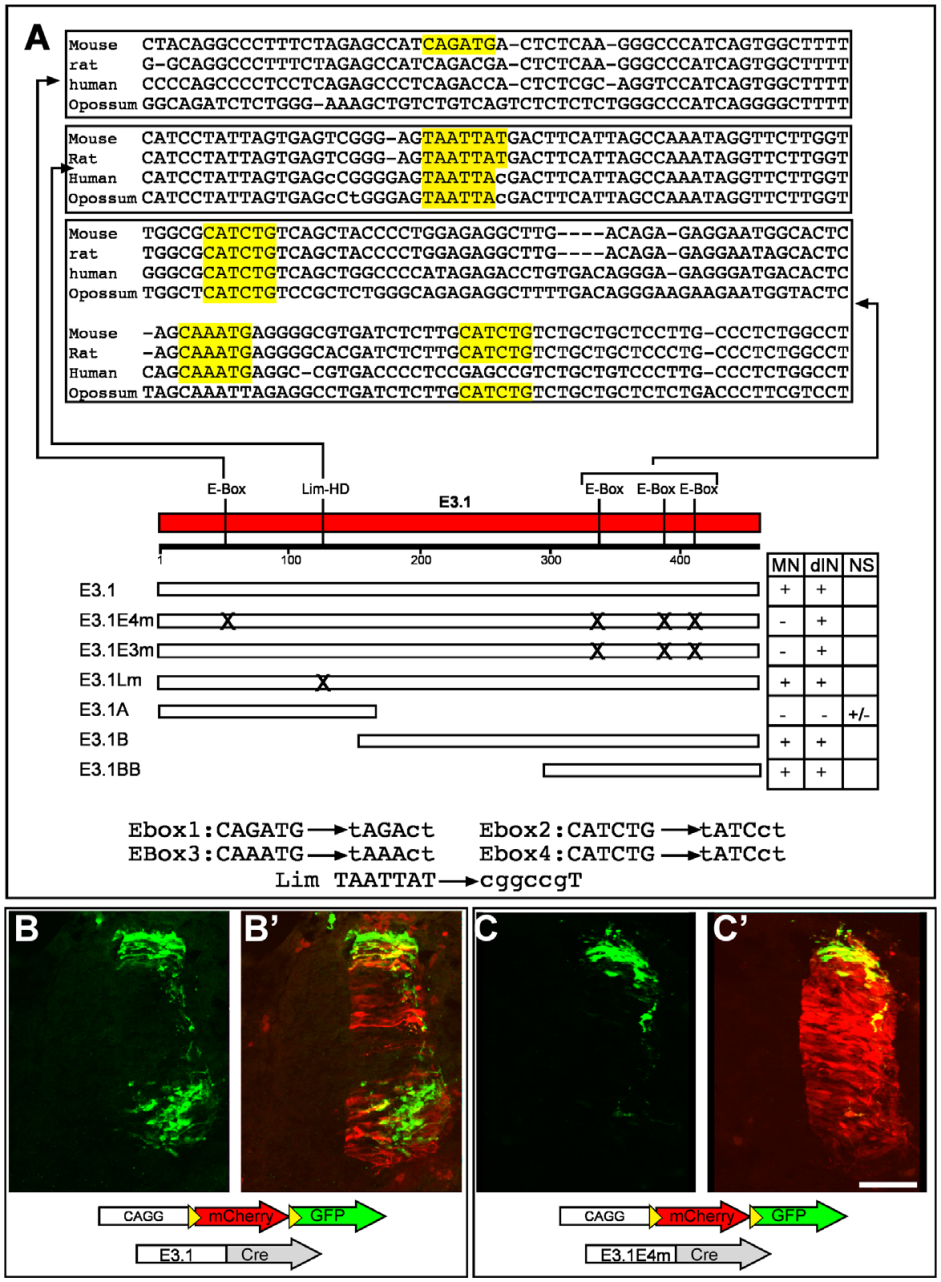
The conserved E-boxes of E3.1 are required for motor neurons expression. A. Sequence alignment of the 5′ E-box and the three 3′ E-boxes; A scheme showing the cis binding sites for bHLH proteins (E-Box) and Lim-HD and a scheme and table of the various deletion and point mutations construct that were used for delineating the expression modules of the E3.1 enhancer element. The table summarizes the expression pattern. MN – motor neurons, dIN – dorsal interneurons, NS – non-specific expression. B,C. The mCherry/GFP alternating reporter plasmid [10] was used to test the activity of the mutated E3.1 enhancer. This plasmid enables the simultaneous detection of the electroporated cells (expressing mCherry) and cells are specifically expressing the enhancer driven reporter (GFP). The non-mutated E3.1 enhancer directed expression to motor neurons and dorsal interneurons, while E3.1 elements that harbors point mutations on all the E-boxes (E3.1E4 m), drives expression only to the dorsal interneurons. Scale Bar A,B 70 µm.

Transcription factors that belong to the bHLH and the Lim-HD families determine the cell fate of spinal neurons [1], [2], [37]. The activity of the E3 enhancer might be stipulated by direct interactions with these transcription factors. The E3 element was screened for potential binding sequences of bHLH and Lim-HD proteins. Four E-boxes, the canonical binding site of bHLH TFs, three of them conserved in mammals and marsupials, and one Lim-HD binding site were found in E3.1 (Fig. 5A), and three E-boxes and one Lim-HD binding sites in E3.3 (not shown). The requirement of the E-boxes and Lim-HD cis element of E3.1 for the expression in the spinal cord was challenged by point mutating the three 3′ conserved E-boxes (designated E3.1E3m), all the four E-boxes (E3.1E4 m) or the Lim-HD binding site (E3.1Lm) (Fig. 5A). E3.1Lm directed expression in an identical pattern to the native E3.1 (not shown), suggesting that the Lim-HD cis element is dispensable for the enhancer properties. Consistently, E3 deletion constructs E3.1B and E3.1BB, which do not contain the Lim-HD target, were sufficient to drive expression in motor and dI1 neurons (Fig. 3F, S4), whereas the complementary element E3.1a that contains only the 5′ E-box and the Lim-HD cis elements yielded only sporadic, non-specific expression (Fig. S4). By contrast, E3.1E4m and E3.1E3m directed expression of a reporter gene to the dorsal interneurons in the same pattern as the native E3.1. Significantly, however, substantially reduced expression from these elements was detected in motor neurons (Fig. 5B,C, table 1, E3.1E3m not shown). The specificity of E3.1, E3.1B, E3.1BB, E3.1E4m and E3.1A was tested by utilizing the alternate mCherry/GFP cassette [10] that directs ubiquitous expression of mCherry and enhancer-mediated expression of GFP (Fig. 4B,C, S4), and immunohistochemistry for TAG1 (Fig. S3). Thus, the motor neuron expression module of the E3 enhancer appears to require bHLH transcription factor binding sites, whereas Lim-HD protein binding sites are dispensable.

### A conserved bHLH target in chick directs expression in motor neurons

**Figure 6:**
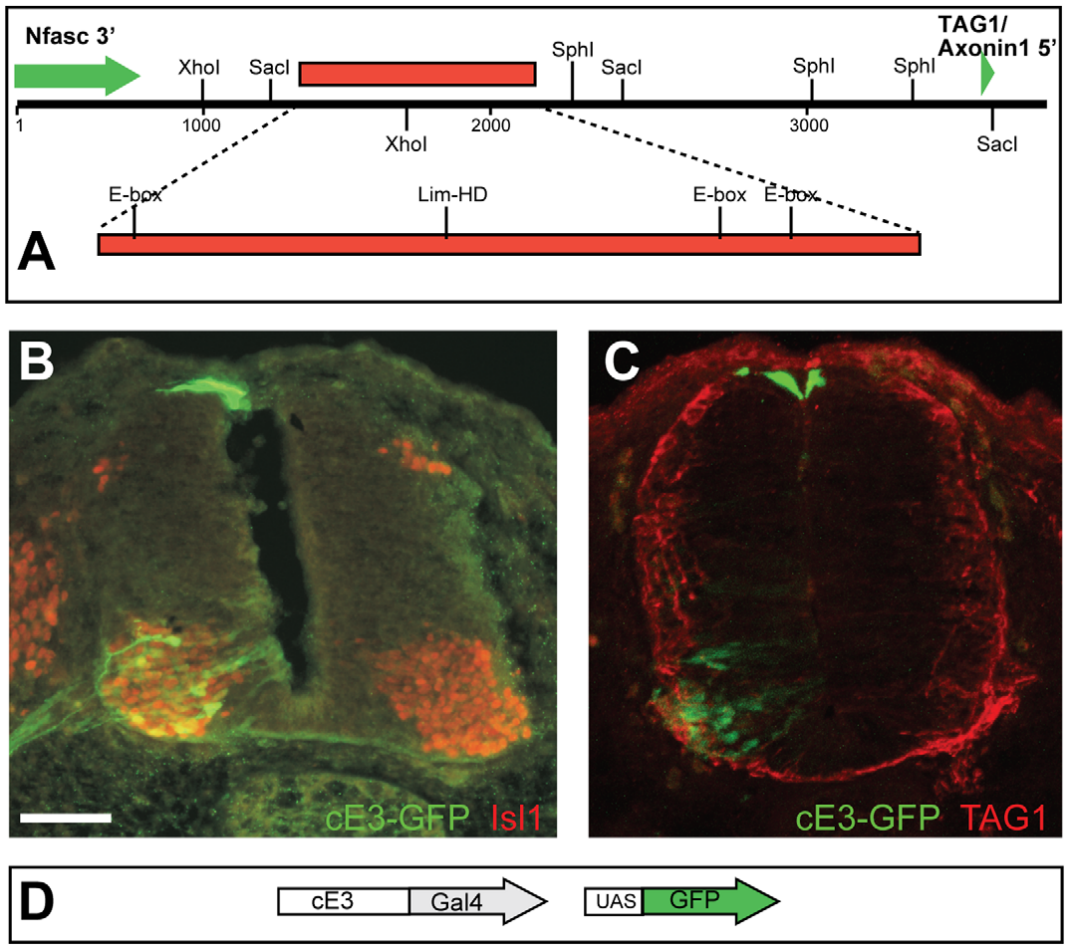
The chick cE enhancer drives expression in motor neurons. A. The genomic organization of the region between the chick *Nfasc* and *TAG1* genes. The red box represents the cE element. B,C. The cE enhancer was cloned upstream to Gal4 transactivator and electroporated at HH18 into the chick neural tube along with UAS-GFP plasmid. GFP is expressed in motor neurons and roof plate cells. B. Co-staining with Isl1 antibody reveals that the ventral GFP expressing neurons express the motor neurons transcription factor Isl1. C. Co-staining with TAG1/Axonin-1 antibody demonstrates that the differentiated motor neurons, reside in the lateral ventral spinal cord, express GFP and TAG1. D. Schematic representation of the plasmids used in B and C. The cE3 enhancer was cloned up stream to Gal4 and co-elecroporated with UAS::GFP plasmid. Scale Bar B–C 50 µm.

Alignment of the genomic region that spans *TAG1* and *Nfasc*, utilizing UCSC alignment tools, revealed that the conservation of TGM-3M and E3 elements is restricted to mammals (Fig. 3A-C, Fig. S1). However, the mouse E3 enhancer drives expression in motor and dorsal interneurons in chick and mouse. Therefore, the trans-activators and the cis elements that mediate expression in MN and dI1 neurons should be conserved between mammals and aves. The 2.7Kb that separate *TAG1* and *Nfasc* genes in the chick were screened for E-boxes. A 760 bp element (cE3 enhancer) that contains three E-boxes was utilized in ovo as a potential enhancer element (Fig. 6A). Two domains of expression are apparent in the ventral spinal cord, 80% of the GFP labeled cells are Isl1+ (Fig. 6B) and TAG1+ (Fig. 6C) indicating expression in motor neurons. In the dorsal spinal cord expression is confined to the dorsal midline. 3% of the GFP labeled cells are Brn3a+ indicating that they are dI1-3 neurons, while 97% of the GFP+/Brn3a- cells reside in the dorsal midline, and are likely to be roof plate cells. Hence, cE3 enhancer directed expression of GFP in motor neurons (Fig. 6A,B), but not in dI1 neurons, thus, providing further support to the hypothesized role of bHLH proteins in regulating the MN expression of the E3 enhancer.

## Discussion

Motor neurons and interneurons emerge from distinct progenitor cell domains along the dorsoventral axis of the neural tube. Progenitor cells in each domain are specified by graded sonic hedgehog (Shh) and BMP signaling, leading to the expression of unique combinations of homeodomain and basic helix-loop-helix (bHLH) transcription factors. Subsequent differentiation and connectivity of spinal neurons is governed by an assortment of cell type specific transcription factors, defined as a transcriptional code, that controls the expression of receptors for guidance cues [1], [2]. TAG1 and Nfasc are co-expressed in numerous neuronal cell types in the CNS and PNS; both promote neurite outgrowth [24], [38], [39] and both are required for the architecture and function of nodes of Ranvier [40], [41]. In addition, TAG1 is required *in vivo* for axon guidance [13], [42], [43], neuronal migration [44], [45], and modulates the responses of sensory axons to diffusible guidance signals by controlling the trafficking of their receptors [43]. In the current study, we have identified two complementary enhancer elements of the *TAG1* gene that direct expression during the mid-embryonic period to either the PNS or the CNS. Further characterization of the CNS enhancer element, situated 3′ to *Nfasc* and 5′ to *TAG1*, demonstrated that it directs expression in dorsal interneurons and in motor neurons. Deletion and point mutation analysis reveal that a minimal element of 130 bp, that contains 3 E-boxes, is required for directing expression in motor neurons.

The genomic link between *TAG1* and *Nfasc* is conserved in all vertebrates: mammals, birds and fish. Their spatial co-expression pattern, in DRG and in commissural and motor neurons, is also conserved (this study; [24], [46], [47], [48]). The preferential E3-mediated expression of reporter genes in the commissural dI1 neurons – dI1c, versus in the ipsilateral subpopulation – dI1i, supports the theory that E3 is a TAG1, rather than Nfasc, enhancer element. However, we cannot exclude that E3 regulates both genes. Deletion of the E3 element in the context of a large BAC that contains both genes, or via gene targeting in mice, would be required to verify this issue.

Our conclusion that regulatory elements lying between 4kb upstream of exon 1 and the translation start site in exon 2 drive expression in the embryonic PNS, appears to be at odds with the report by Denaxa et al that a fragment lying 4 kb 5′ to exon 1 directs expression to commissural axons [27]. However, although there is ambiguity as to exactly which elements were assayed in this study (the 4 kb sequence deposited in Genbank, X92681, appears to be compilation of sequences from different parts of the CNTN2 locus), an apparent difference is that their construct did not include sequences from intron 1, which is known to harbor key regulatory elements in the genes of other members of the TAG1 family [28], [29]. Notably all of the constructs we assayed included at least the first 2 kb of intron 1, which is enriched in conserved sequences, yet in none of these constructs did we see expression within the neural tube, suggesting this region may contain elements that suppress CNS expression in the embryo. Moreover, the most robust expression was found when all of intron 1 was present, suggesting that further intronic elements contribute to the complete embryonic PNS expression pattern.

The separation of regulatory elements controlling PNS from CNS expression might suggest that these different systems may have evolved separately. However, this separation may be restricted to the mid-embryonic phase, since at later stages the TGM-3M enhancer also drives expression in the CNS, notably in the cerebellum [26], the cortex [49] and the retina (SVK unpublished).

The E3 element was isolated based on its homology across mammals. Yet, the mouse E3 element directs expression of reporter gene to motor neurons and dorsal interneurons in both mouse and chick. These observations are highly indicative that the transcriptional complexes directing the expression in these two neuronal populations are potentially conserved among vertebrates. It is also plausible that, through evolution, only the obligatory short cis elements, e.g. the E-boxes, remained conserved between mammals and avian.

bHLH and Lim-HD proteins are expressed in motor and dI1 neurons: Olig2, Ngn2, NeuroD and Ascl1 in motor neurons and Atoh1 in dI1 neurons; Isl1,Isl2 and Lhx3 in motor neurons and Lhx2 and Lhx9 in dI1 neurons. Ectopic expression of the dI1 transcription factors –Lhx2 and Lhx9, either separately or in combination, failed to elevate the expression of the endogenous TAG1 (not shown). It is likely, therefore, that other transcription factors play a role in regulating the expression of TAG1 in commissural interneurons. Two candidates were tested by us: BarHl1 (expressed in dI1 neurons) and UNC4 (expressed in all the commissural INs) – neither of these was sufficient to up-regulate the expression of TAG1 (data not shown).

Among the dorsal commissural interneurons, E3 directed expression only in dI1 neurons. Based on the expression of TAG1 and Robo3, dI2, dI4 and dI6 are also commissural neurons [5], [10]. All these interneurons express bHLH and Lim-HD proteins: Atoh1+Lhx2/9 in dI1, Ngn1/2+Lhx1/5 in dI2,dI4 and dI6. The combined expression of bHLH and Lim-HD proteins is probably not the sole requirement for mediating the expression of commissural specific receptors for guidance cues, such as Robo3 and TAG1, since the ipsi-lateral projecting INs (dI1i and dI3) express a similar combination of bHLH and Lim-HD TFs (Lhx9+Atoh1 and Isl1+Ngn2, respectively), yet do not express either Robo3 or TAG1 [5]. Hence, a higher combinatorial order of transcription factors, presumably including additional proteins, is likely to control the expression of commissural neuron guidance cue receptors.

The identity of motor neurons is traditionally monitored by the expression of transcription factors that are expressed along the differentiation cascade, such as Isl1 and Hb9 [50], [51]. In such studies, various bHLH proteins have been found to be involved in the neurogenesis and differentiation of motor neurons. Olig2 is required to determine the progenitor motor neuron (pMN) fate. Functional studies indicate that Olig2 down-regulation is required to release pMN cells from an inhibitory block on post-mitotic motor neuron formation [52], [53], [54]. An Olig2 to Ngn2 switch is required for the differentiation of post-mitotic motor neurons [52]. In addition, the coordinated activity of Lim-HD proteins Isl1 and Lhx3, with bHLH proteins, either NeuroM or Ngn2, synchronizes the neurogenesis and specification of motor neurons [50]. In the current study, the expression of a known guidance-cue receptor – TAG1 – was studied. The sole requirement of the E-boxes to recapitulate the endogenous expression in the mid-gestation spinal cord, suggests that post-neurogenesis, a bHLH protein may function as a critical transcription factor regulating the expression of axon guidance receptors in motor neurons.

## Materials and Methods

### In ovo electroporations

Fertilized white Leghorn chicken eggs were incubated at 38.5 to 39°C. A DNA solution of 5 mg/ml was injected into the lumen of the neural tube at stage HH17 to HH18. Electroporation was performed using three 50 ms pulses at 25V, applied across the embryo using a 0.5 mm Tungsten wire and a BTX electroporator (ECM 830). Embryos were incubated for additional 1 to 3 days prior to analysis.

### Immunohistochemistry

Embryos were fixed overnight at 4°C in 4% paraformaldehyde/0.1 M phosphate buffer, washed twice with phosphate-buffered saline, incubated in 30% sucrose/phosphate-buffered saline for 24 h, and embedded in Optimal Cutting Temperature solution (OCT). Cryostat sections (14 µm) were collected on Superfrost Plus slides and kept at −70°C. The following antibodies were used: rabbit polyclonal anti GFP antibody (Molecular Probes, Eugene, Oregon, USA), Mouse anti GFP (Chemicon, Temecula, CA, USA), Rabbit anti RFP (Chemicon, Temecula, CA, USA), Isl1 (4D5), Lhx1/5 (4F2), Lhx2/9 (rabbit serum) (provided by T. Jessell, Columbia University, New York, NY, USA). Axonin1 (rabbit serum, provided by Esther Stoeckli), Nfasc antibody (provided by Fritz G. Rathjen). Mouse anti Brn3a (LifeSpan BioSciences) Cy2, RRX and cy5 were used as fluorochromes. Images were taken under a microscope (Axioscope 2; Zeiss) with a digital camera (DP70; Olympus) or confocal microscope (FV1000; Olympus).

### DNA

The mouse E3 enhancer element and it short fragments were amplified by PCR from a genomic mouse DNA utilizing the following primers:

E3- [TACCCAATCCTTTCCACTTCC] and [GCTTGTCAACCTTTGAGTCAAG]

E3.1-[TACCCAATCCTTTCCACTTCC] and [ATGACTGGGAAGGTGAACCA]

E3.1a-[TACCCAATCCTTTCCACTTCC] and [GAGAGTCTCTGAACCAAGAAC]

E3.1b-[GTTCTTGGTTCAGAGACTCTC] and [ATGACTGGGAAGGTGAACCA]

E3.1bb-[AGAAAGAGGCTCCCTGCCCTC] and [AGAGGGCAAGGAGCAGCAGAC]

E3.3--[AAGATGCCATCTGCTGGGCTGAG] and [AGAGAGGCTCCTGGCACTTT]

The 1^st^ (5′) E Box mutation was generated by using the following primers:

[TACCCAATCCTTTCCACTTCCTGCTCTACAGGCCCTTTCTAGAGCCATtAGActACTCTCAAGGGCCCATC] and [ATGACTGGGAAGGTGAACCA].

The 2^nd^–4^th^ E Box mutations were generated by two-step PCR reactions. For each mutation two complementary primers, containing the mutation and 15 bp (3′ 15 bp and 5′ 15 bp) flanking sequences were designed. In the 1^st^ step two PCR reactions were done: with the E3.1 5′ primer (see above) plus the 3′ mutation primer and the 5′ mutation primer plus the E3.1 3′ primer. The products of the PCR reactions were mixed and a 2^nd^ step PCR reaction was preformed with the 5′ and 3′ E3.1 primers. The following primers, and their complementary primers, were designed for the mutations:

2^nd^ E-Box: [CTCCTCCGTGGCGtATCctTCAGCTACCCCTGGAG]; 3^rd^ E-Box: [GGAATGGCACTCAGtAAActAGGGGCGTGATCTC]; 4^th^ E-Box: [CGTGATCTCTTGtATCctTCTGCTGCTCCTTGC].

Similar strategy was utilized for the Lim-HD mutation using [GAGTCGGGAGcggccgtgaCTTCATTAG]

The chick E3 enhancer element was amplified by PCR from a genomic chick DNA utilizing the following primers: [CTGCCTAACACCATCCTTGC] and [CATCTATCAAAGCATTCAG].

SacI and PacI sites were introduced to the 5′ and 3′ primers, respectively. The PCR products were subcloned into either Cre or Gal4 plasmids [9], [10]. The target plasmids for the Cre and Gal4 drivers were described [9], [10].

### Transgenic mice

Elements of E3.1 enhancer fragment, or a mutant version thereof lacking E-box binding sites, have been cloned upstream of a minimal Hsp68 promoter driving expression of LacZ marker. Purified fragments have been injected in fertilized mouse oocytes (FVB strain) and transferred into pseudopregnant recipient females by standard procedures. Embryos were collected at appropriate gestation age (mostly, at E11.5 post fertilization), yolk sacs separated for subsequent genotyping to determine transgene integration while embryos were fixed in 1% formaline/0.25% glutaraldehyde. Staining in 1mg/ml X-gal solution, also containing 2 mM MgCl_2_, 0.05% Triton-X100 and 5 mM each of K_4_[Fe(CN)_6_] and K_3_[Fe(CN)_6_] at room temperature has been allowed to proceed overnight.

## Supporting Information

Figure S1 **The organization and conservation of the** ***TAG1*** **gene.** Scheme showing organization of mouse *TAG1* gene 7 kb upstream and 8.5 kb downstream of exon 1. Blue boxes directly below indicate the sequences of the human *TAG1* gene deposited in Genbank by Denaxa et al., 2003 (Accession Number X92681.1), from which substantial parts of intron 1 and intron 2 are missing (dashed lines). Below these are indicated constructs used in this study, which include human *TAG1* genomic DNA (blue line) fused in-frame at the ATG codon to a LacZ reporter gene (light blue). Below that, an alignment of the mouse region to rat, rabbit, dog, elephant and opossum elements. The Alignment was done using BLAT alignment tool of UCSC genome bioinformatics (http://genome.ucsc.edu/cgi-bin/hgGateway).
(TIF)

Figure S2 **E3 enhancer direct expression preferentially to dI1c neurons.** Co expression of GFP driven by dI1 specific enhancer [10], and mCherry driven by E3 enhancer (E). A-D Images of the ventral lateral spinal cord. Yellow arrowheads point to the dorsal medial dI1 neurons (the position occupied by dI1c neurons) that are co-labeled by GFP and mcherry. Green arrowheads point to the ventral lateral dI1 neurons (the position occupied by dI1i neurons) that are labeled by EdI1-derived GFP, but not mcherry-derived E3. Yellow arrows point to commissural axons that project toward or at the floor plate. Green arrows point to ipsilaterally projecting axons. Red arrows point to motor axons. Scale Bar in D 100 µm.
(TIF)

Figure S3 **Co-expression of enhancer driven GFP and TAG1/Axonin-1 and Isl1 proteins.** The E3.1 (A,D), E3.3 (B,E) and cE3 (C,F) were used to drive expression of GFP (A–C) or nFGP (D-F) in the chick spinal cord. Cross sections of HH24 were co-stained with Axonin-1 (A–C) and Isl1 (D–F) antibodies. The images in A-C are the double stained sections shown in Fig. 3D-G, respectively. The arrows point to neurons that co-express GFP and Axonin-1. Note that the GFP+/Axonin- neurons (green arrows) are positioned in the ventricular zone and the medial spinal cord, a position occupied by progenitor neurons. Thus, GFP+/Axonin- neurons are likely progenitor motor neurons (pMN) and progenitor dI1 neurons (pdI1) that have not initiate the expression of the TAG1/Axonin. Scale Bar in C – 50 µm.
(TIF)

Figure S4 **Specificity analysis of enhancer elements.** The mCherry/GFP alternating reporter plasmid (E) [10] was used to test the activity of E3 (A), E3.1B (B), E3.1BB (C) and E3.1A (D) enhancers. This plasmid enables the simultaneous detection of the electroporated cells (expressing mCherry) and cells specifically expressing the enhancer driven reporter (GFP). Ubiquitous expression of mcherry is demonstrated in cross sections of HH24 spinal cords, while expression of GFP in E3, E3.1B and E3.1BB is restricted to motor neurons and dorsal interneurons. E3.1A directs sporadic and non-specific expression of GFP (D). Scale Bar in C – 50 µm.
(TIF)

## References

[1] Shirasaki R, Pfaff SL (2002) Transcriptional codes and the control of neuronal identity. Annu Rev Neurosci 25: 251–281. 12052910 10.1146/annurev.neuro.25.112701.142916

[2] Jessell TM (2000) Neuronal specification in the spinal cord: inductive signals and transcriptional codes. Nat Rev Genet 1: 20–29. 11262869 10.1038/35049541

[3] Kania A, Jessell TM (2003) Topographic motor projections in the limb imposed by LIM homeodomain protein regulation of ephrin-A:EphA interactions. Neuron 38: 581–596. 12765610 10.1016/s0896-6273(03)00292-7

[4] Luria V, Krawchuk D, Jessell TM, Laufer E, Kania A (2008) Specification of motor axon trajectory by ephrin-B:EphB signaling: symmetrical control of axonal patterning in the developing limb. NEURON 60: 1039–1053. 19109910 10.1016/j.neuron.2008.11.011

[5] Wilson SI, Shafer B, Lee KJ, Dodd J (2008) A molecular program for contralateral trajectory: Rig-1 control by LIM homeodomain transcription factors. Neuron 59: 413–424. 18701067 10.1016/j.neuron.2008.07.020

[6] Lance-Jones C, Landmesser L (1980) Motoneurone projection patterns in the chick hind limb following early partial reversals of the spinal cord. The Journal of physiology 302: 581–602. 7411470 10.1113/jphysiol.1980.sp013262 PMC1282866

[7] Kania A, Johnson RL, Jessell TM (2000) Coordinate roles for LIM homeobox genes in directing the dorsoventral trajectory of motor axons in the vertebrate limb. Cell 102: 161–173. 10943837 10.1016/s0092-8674(00)00022-2

[8] Dasen JS, Tice BC, Brenner-Morton S, Jessell TM (2005) A Hox regulatory network establishes motor neuron pool identity and target-muscle connectivity. Cell 123: 477–491. 16269338 10.1016/j.cell.2005.09.009

[9] Avraham O, Hadas Y, Vald L, Hong S, Song MR, et al (2010) Motor and Dorsal Root Ganglion Axons Serve as Choice Points for the Ipsilateral Turning of dI3 Axons. J Neurosci 30: 15546–15557. 21084609 10.1523/JNEUROSCI.2380-10.2010 PMC6633670

[10] Avraham O, Hadas Y, Vald L, Zisman S, Schejter A, et al (2009) Transcriptional control of axonal guidance and sorting in dorsal interneurons by the Lim-HD proteins Lhx9 and Lhx1. Neural Dev 4: 21. 19545367 10.1186/1749-8104-4-21 PMC2704203

[11] Crone SA, Quinlan KA, Zagoraiou L, Droho S, Restrepo CE, et al (2008) Genetic ablation of V2a ipsilateral interneurons disrupts left-right locomotor coordination in mammalian spinal cord. Neuron 60: 70–83. 18940589 10.1016/j.neuron.2008.08.009

[12] Dickson BJ, Zou Y (2010) Navigating intermediate targets: the nervous system midline. Cold Spring Harbor perspectives in biology 2: a002055. 20534708 10.1101/cshperspect.a002055 PMC2908764

[13] Stoeckli ET, Landmesser LT (1995) Axonin-1, Nr-CAM, and Ng-CAM play different roles in the in vivo guidance of chick commissural neurons. Neuron 14: 1165–1179. 7541632 10.1016/0896-6273(95)90264-3

[14] Fitzli D, Stoeckli ET, Kunz S, Siribour K, Rader C, et al (2000) A direct interaction of axonin-1 with NgCAM-related cell adhesion molecule (NrCAM) results in guidance, but not growth of commissural axons. The Journal of cell biology 149: 951–968. 10811834 10.1083/jcb.149.4.951 PMC2174557

[15] Jaworski A, Long H, Tessier-Lavigne M (2010) Collaborative and specialized functions of Robo1 and Robo2 in spinal commissural axon guidance. The Journal of neuroscience: the official journal of the Society for Neuroscience 30: 9445–9453. 20631173 10.1523/JNEUROSCI.6290-09.2010 PMC6632452

[16] Long H, Sabatier C, Ma L, Plump A, Yuan W, et al (2004) Conserved roles for Slit and Robo proteins in midline commissural axon guidance. Neuron 42: 213–223. 15091338 10.1016/s0896-6273(04)00179-5

[17] Garcia-Frigola C, Carreres MI, Vegar C, Mason C, Herrera E (2008) Zic2 promotes axonal divergence at the optic chiasm midline by EphB1-dependent and -independent mechanisms. Development (Cambridge, England) 135: 1833–1841. 10.1242/dev.020693 18417618

[18] Ding Q, Joshi PS, Xie Z-H, Xiang M, Gan L (2012) BARHL2 transcription factor regulates the ipsilateral/contralateral subtype divergence in postmitotic dI1 neurons of the developing spinal cord. Proceedings of the National Academy of Sciences of the United States of America 109: 1566–1571. 22307612 10.1073/pnas.1112392109 PMC3277160

[19] Avraham O, Zisman S, Hadas Y, Vald L, Klar A (2010) Deciphering axonal pathways of genetically defined groups of neurons in the chick neural tube utilizing in ovo electroporation. J Vis Exp. 10.3791/1792 PMC2902355 20440258

[20] Marcos-Mondejar P, Peregrin S, Li JY, Carlsson L, Tole S, et al (2012) The lhx2 transcription factor controls thalamocortical axonal guidance by specific regulation of robo1 and robo2 receptors. The Journal of neuroscience: the official journal of the Society for Neuroscience 32: 4372–4385. 22457488 10.1523/JNEUROSCI.5851-11.2012 PMC6622047

[21] Stoeckli ET, Kuhn TB, Duc CO, Ruegg MA, Sonderegger P (1991) The axonally secreted protein axonin-1 is a potent substratum for neurite growth. The Journal of cell biology 112: 449–455. 1991792 10.1083/jcb.112.3.449 PMC2288832

[22] Wolfer DP, Giger RJ, Stagliar M, Sonderegger P, Lipp HP (1998) Expression of the axon growth-related neural adhesion molecule TAG-1/axonin-1 in the adult mouse brain. Anatomy and embryology 197: 177–185. 9543336 10.1007/s004290050129

[23] Dodd J, Morton SB, Karagogeos D, Yamamoto M, Jessell TM (1988) Spatial regulation of axonal glycoprotein expression on subsets of embryonic spinal neurons. Neuron 1: 105–116. 3272160 10.1016/0896-6273(88)90194-8

[24] Furley AJ, Morton SB, Manalo D, Karagogeos D, Dodd J, et al (1990) The axonal glycoprotein TAG-1 is an immunoglobulin superfamily member with neurite outgrowth-promoting activity. Cell 61: 157–170. 2317872 10.1016/0092-8674(90)90223-2

[25] Karagogeos D, Morton SB, Casano F, Dodd J, Jessell TM (1991) Developmental expression of the axonal glycoprotein TAG-1: differential regulation by central and peripheral neurons in vitro. Development (Cambridge, England) 112: 51–67. 10.1242/dev.112.1.51 1769341

[26] Bizzoca A, Virgintino D, Lorusso L, Buttiglione M, Yoshida L, et al (2003) Transgenic mice expressing F3/contactin from the TAG-1 promoter exhibit developmentally regulated changes in the differentiation of cerebellar neurons. Development (Cambridge, England) 130: 29–43. 10.1242/dev.00183 12441289

[27] Denaxa M, Pavlou O, Tsiotra P, Papadopoulos GC, Liapaki K, et al (2003) The upstream regulatory region of the gene for the human homologue of the adhesion molecule TAG-1 contains elements driving neural specific expression in vivo. Brain research Molecular brain research 118: 91–101. 14559358 10.1016/j.molbrainres.2003.07.004

[28] Cangiano G, Ambrosini M, Patruno A, Tino A, Buttiglione M, et al (1997) Functional organization of the promoter region of the mouse F3 axonal glycoprotein gene. Brain research Molecular brain research 48: 279–290. 9332725 10.1016/s0169-328x(97)00100-9

[29] Jones FS, Burgoon MP, Hoffman S, Crossin KL, Cunningham BA, et al (1988) A cDNA clone for cytotactin contains sequences similar to epidermal growth factor-like repeats and segments of fibronectin and fibrinogen. Proceedings of the National Academy of Sciences of the United States of America 85: 2186–2190. 2451243 10.1073/pnas.85.7.2186 PMC279954

[30] Brummendorf T, Rathjen FG (1996) Structure/function relationships of axon-associated adhesion receptors of the immunoglobulin superfamily. Current opinion in neurobiology 6: 584–593. 8937821 10.1016/s0959-4388(96)80089-4

[31] Shiga T, Oppenheim RW (1991) Immunolocalization studies of putative guidance molecules used by axons and growth cones of intersegemental interneurons in the chick embryo spinal cord. The Journal of comparative neurology 310: 234–252. 1720141 10.1002/cne.903100208

[32] Pennacchio LA, Ahituv N, Moses AM, Prabhakar S, Nobrega MA, et al (2006) In vivo enhancer analysis of human conserved non-coding sequences. Nature 444: 499–502. 17086198 10.1038/nature05295

[33] Visel A, Bristow J, Pennacchio LA (2007) Enhancer identification through comparative genomics. Semin Cell Dev Biol 18: 140–152. 17276707 10.1016/j.semcdb.2006.12.014 PMC1855162

[34] Visel A, Minovitsky S, Dubchak I, Pennacchio LA (2007) VISTA Enhancer Browser--a database of tissue-specific human enhancers. Nucleic Acids Res 35: D88–92. 17130149 10.1093/nar/gkl822 PMC1716724

[35] Suster ML, Kania A, Liao M, Asakawa K, Charron F, et al (2009) A novel conserved evx1 enhancer links spinal interneuron morphology and cis-regulation from fish to mammals. Developmental biology 325: 422–433. 18992237 10.1016/j.ydbio.2008.10.004

[36] Mansour AA, Nissim-Eliraz E, Zisman S, Golan-Lev T, Schatz O, et al (2011) Foxa2 regulates the expression of Nato3 in the floor plate by a novel evolutionarily conserved promoter. Mol Cell Neurosci 46: 187–199. 20849957 10.1016/j.mcn.2010.09.002

[37] Helms AW, Johnson JE (2003) Specification of dorsal spinal cord interneurons. Curr Opin Neurobiol 13: 42–49. 12593981 10.1016/s0959-4388(03)00010-2

[38] Felsenfeld DP, Hynes MA, Skoler KM, Furley AJ, Jessell TM (1994) TAG-1 can mediate homophilic binding, but neurite outgrowth on TAG-1 requires an L1-like molecule and beta 1 integrins. Neuron 12: 675–690. 7512353 10.1016/0896-6273(94)90222-4

[39] Volkmer H, Leuschner R, Zacharias U, Rathjen FG (1996) Neurofascin induces neurites by heterophilic interactions with axonal NrCAM while NrCAM requires F11 on the axonal surface to extend neurites. The Journal of cell biology 135: 1059–1069. 8922386 10.1083/jcb.135.4.1059 PMC2133392

[40] Gollan L, Salomon D, Salzer JL, Peles E (2003) Caspr regulates the processing of contactin and inhibits its binding to neurofascin. The Journal of cell biology 163: 1213–1218. 14676309 10.1083/jcb.200309147 PMC2173730

[41] Poliak S, Salomon D, Elhanany H, Sabanay H, Kiernan B, et al (2003) Juxtaparanodal clustering of Shaker-like K+ channels in myelinated axons depends on Caspr2 and TAG-1. The Journal of cell biology 162: 1149–1160. 12963709 10.1083/jcb.200305018 PMC2172860

[42] Perrin FE, Rathjen FG, Stoeckli ET (2001) Distinct subpopulations of sensory afferents require F11 or axonin-1 for growth to their target layers within the spinal cord of the chick. Neuron 30: 707–723. 11430805 10.1016/s0896-6273(01)00315-4

[43] Law CO, Kirby RJ, Aghamohammadzadeh S, Furley AJW (2008) The neural adhesion molecule TAG-1 modulates responses of sensory axons to diffusible guidance signals. Development (Cambridge, England) 135: 2361–2371. 10.1242/dev.009019 18550718

[44] Denaxa M, Kyriakopoulou K, Theodorakis K, Trichas G, Vidaki M, et al (2005) The adhesion molecule TAG-1 is required for proper migration of the superficial migratory stream in the medulla but not of cortical interneurons. Developmental biology 288: 87–99. 16225856 10.1016/j.ydbio.2005.09.021

[45] Sittaramane V, Sawant A, Wolman MA, Maves L, Halloran MC, et al (2009) The cell adhesion molecule Tag1, transmembrane protein Stbm/Vangl2, and Lamininalpha1 exhibit genetic interactions during migration of facial branchiomotor neurons in zebrafish. Developmental biology 325: 363–373. 19013446 10.1016/j.ydbio.2008.10.030 PMC2991145

[46] Rathjen FG, Rutishauser U (1984) Comparison of two cell surface molecules involved in neural cell adhesion. The EMBO journal 3: 461–465. 6201360 10.1002/j.1460-2075.1984.tb01828.x PMC557366

[47] Ruegg MA, Stoeckli ET, Lanz RB, Streit P, Sonderegger P (1989) A homologue of the axonally secreted protein axonin-1 is an integral membrane protein of nerve fiber tracts involved in neurite fasciculation. The Journal of cell biology 109: 2363–2378. 2509484 10.1083/jcb.109.5.2363 PMC2115876

[48] Lustig M, Zanazzi G, Sakurai T, Blanco C, Levinson SR, et al (2001) Nr-CAM and neurofascin interactions regulate ankyrin G and sodium channel clustering at the node of Ranvier. Current biology: CB 11: 1864–1869. 11728309 10.1016/s0960-9822(01)00586-3

[49] Bizzoca A, Corsi P, Polizzi A, Pinto MF, Xenaki D, et al (2012) F3/Contactin acts as a modulator of neurogenesis during cerebral cortex development. Developmental biology 365: 133–151. 22360968 10.1016/j.ydbio.2012.02.011

[50] Lee SK, Pfaff SL (2003) Synchronization of neurogenesis and motor neuron specification by direct coupling of bHLH and homeodomain transcription factors. Neuron 38: 731–745. 12797958 10.1016/s0896-6273(03)00296-4

[51] Song M-R, Sun Y, Bryson A, Gill GN, Evans SM, et al (2009) Islet-to-LMO stoichiometries control the function of transcription complexes that specify motor neuron and V2a interneuron identity. Development (Cambridge, England) 136: 2923–2932. 10.1242/dev.037986 PMC2723064 19666821

[52] Lee S-K, Lee B, Ruiz EC, Pfaff SL (2005) Olig2 and Ngn2 function in opposition to modulate gene expression in motor neuron progenitor cells. Genes & development 19: 282–294. 15655114 10.1101/gad.1257105 PMC545894

[53] Mizuguchi R, Sugimori M, Takebayashi H, Kosako H, Nagao M, et al (2001) Combinatorial roles of olig2 and neurogenin2 in the coordinated induction of pan-neuronal and subtype-specific properties of motoneurons. Neuron 31: 757–771. 11567615 10.1016/s0896-6273(01)00413-5

[54] Novitch BG, Chen AI, Jessell TM (2001) Coordinate regulation of motor neuron subtype identity and pan-neuronal properties by the bHLH repressor Olig2. Neuron 31: 773–789. 11567616 10.1016/s0896-6273(01)00407-x

