# Supplementary figures and images for: Distinct Cis Regulatory Elements Govern the Expression of TAG1 in Embryonic Sensory Ganglia and Spinal Cord
Source: PLoS One. 2013 Feb 26;8(2):e57960. doi: 10.1371/journal.pone.0057960 (PMC3582508; doi:10.1371/journal.pone.0057960)

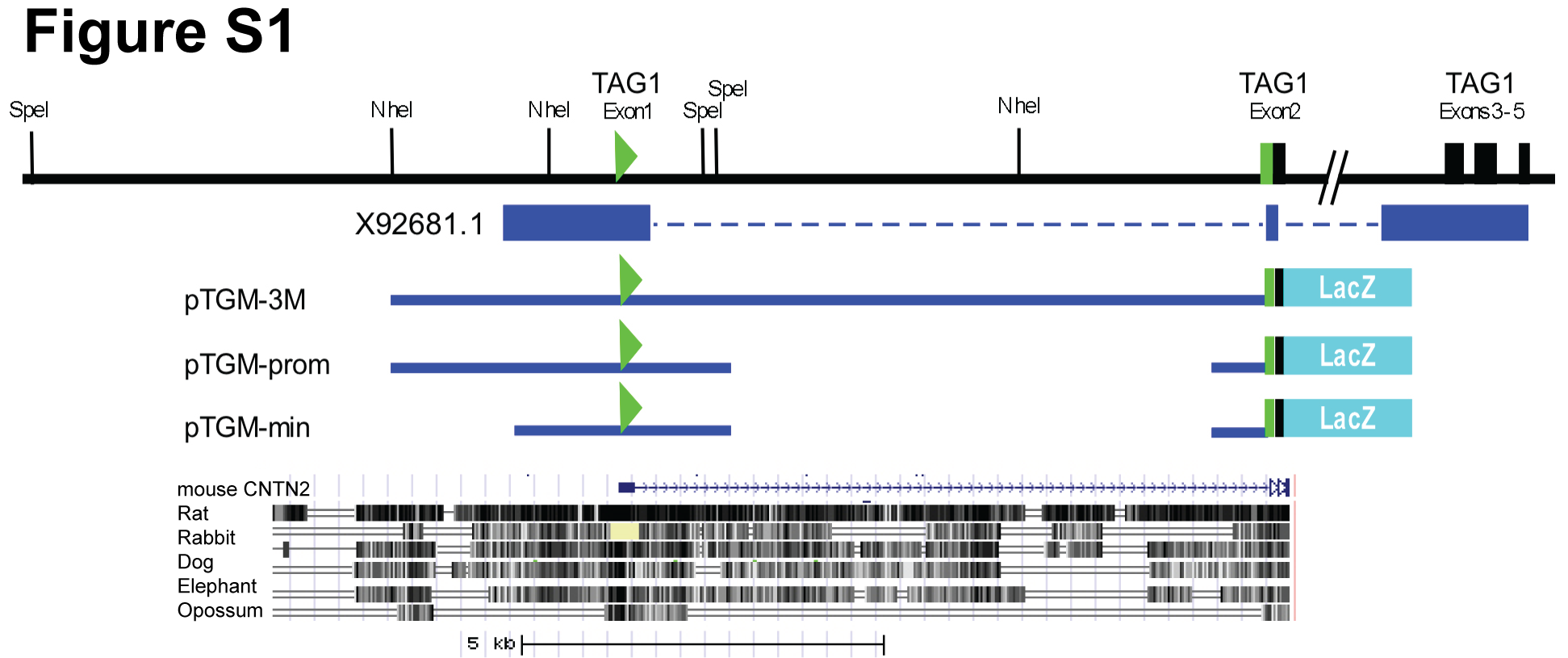

Supplement: Figure S1 — The organization and conservation of the TAG1 gene. Scheme showing organization of mouse TAG1 gene 7 kb upstream and 8.5 kb downstream of exon 1. Blue boxes directly below indicate the sequences of the human TAG1 gene deposited in Genbank by Denaxa et al., 2003 (Accession Number X92681.1), from which substantial parts of intron 1 and intron 2 are missing (dashed lines). Below these are indicated constructs used in this study, which include human TAG1 genomic DNA (blue line) fused in-frame at the ATG codon to a LacZ reporter gene (light blue). Below that, an alignment of the mouse region to rat, rabbit, dog, elephant and opossum elements. The Alignment was done using BLAT alignment tool of UCSC genome bioinformatics (http://genome.ucsc.edu/cgi-bin/hgGateway). (TIF) [file pone.0057960.s001.tif]

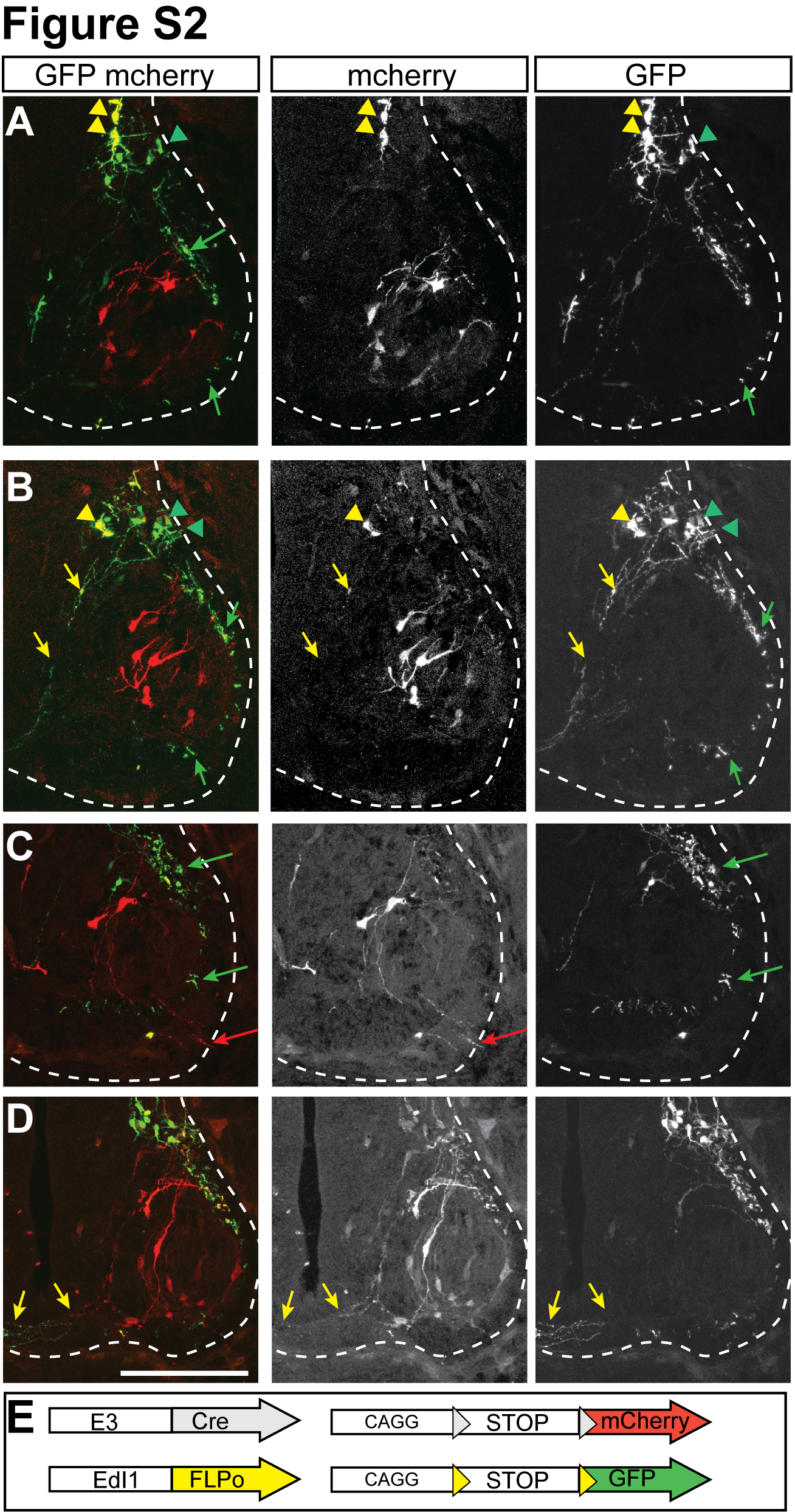

Supplement: Figure S2 — E3 enhancer direct expression preferentially to dI1c neurons. Co expression of GFP driven by dI1 specific enhancer [10], and mCherry driven by E3 enhancer (E). A-D Images of the ventral lateral spinal cord. Yellow arrowheads point to the dorsal medial dI1 neurons (the position occupied by dI1c neurons) that are co-labeled by GFP and mcherry. Green arrowheads point to the ventral lateral dI1 neurons (the position occupied by dI1i neurons) that are labeled by EdI1-derived GFP, but not mcherry-derived E3. Yellow arrows point to commissural axons that project toward or at the floor plate. Green arrows point to ipsilaterally projecting axons. Red arrows point to motor axons. Scale Bar in D 100 µm. (TIF) [file pone.0057960.s002.tif]

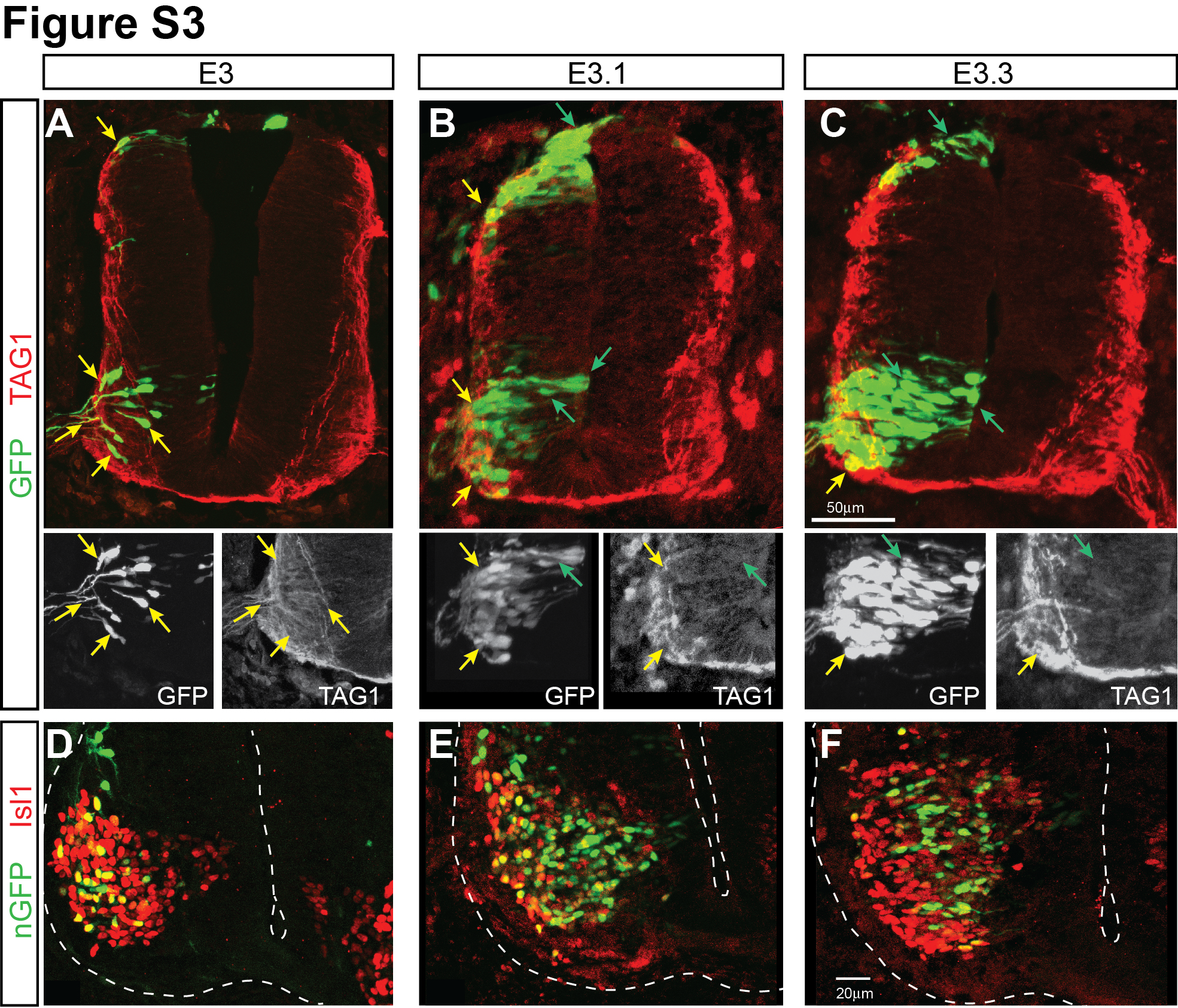

Supplement: Figure S3 — Co-expression of enhancer driven GFP and TAG1/Axonin-1 and Isl1 proteins. The E3.1 (A,D), E3.3 (B,E) and cE3 (C,F) were used to drive expression of GFP (A–C) or nFGP (D-F) in the chick spinal cord. Cross sections of HH24 were co-stained with Axonin-1 (A–C) and Isl1 (D–F) antibodies. The images in A-C are the double stained sections shown in Fig. 3D-G, respectively. The arrows point to neurons that co-express GFP and Axonin-1. Note that the GFP+/Axonin- neurons (green arrows) are positioned in the ventricular zone and the medial spinal cord, a position occupied by progenitor neurons. Thus, GFP+/Axonin- neurons are likely progenitor motor neurons (pMN) and progenitor dI1 neurons (pdI1) that have not initiate the expression of the TAG1/Axonin. Scale Bar in C – 50 µm. (TIF) [file pone.0057960.s003.tif]

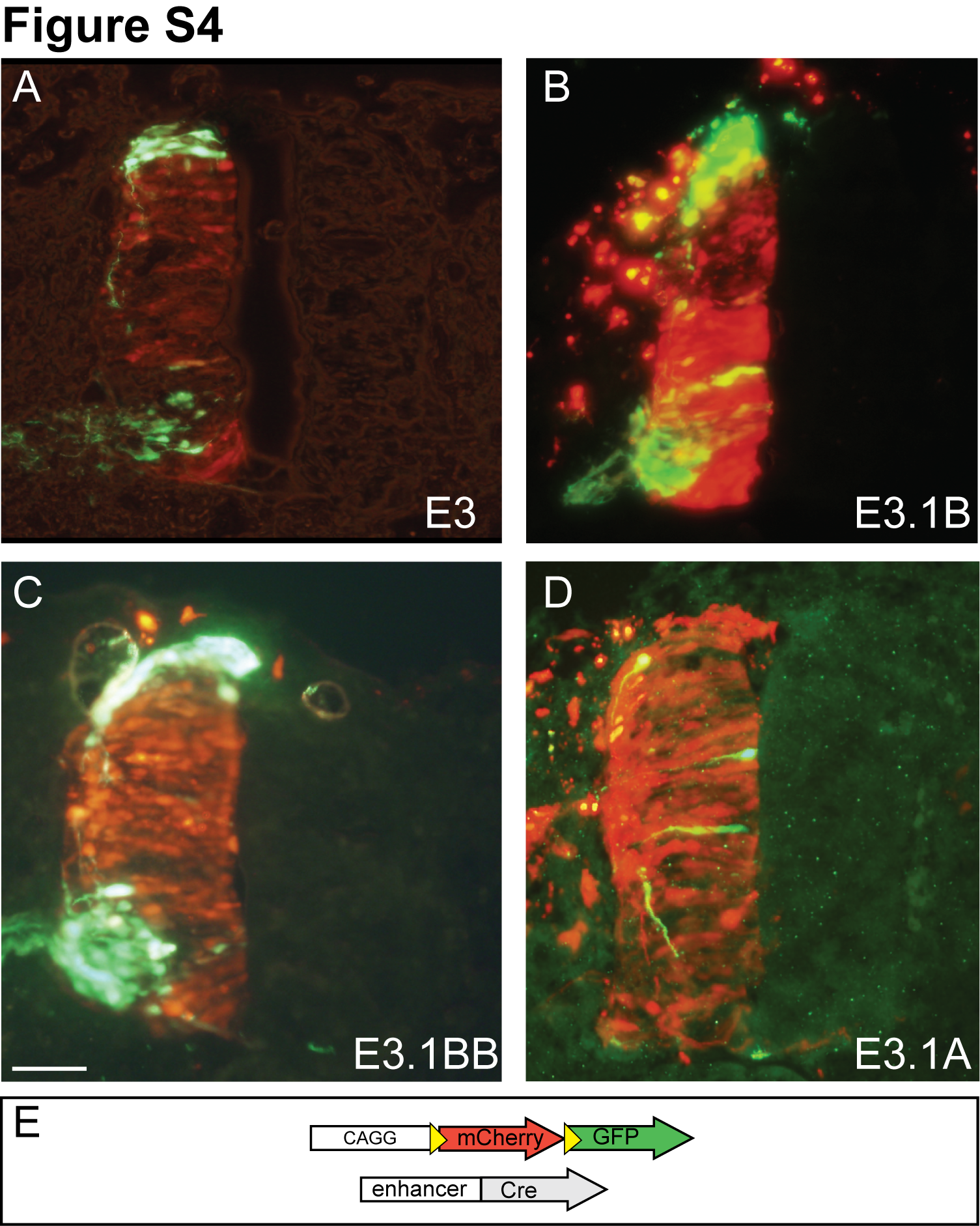

Supplement: Figure S4 — Specificity analysis of enhancer elements. The mCherry/GFP alternating reporter plasmid (E) [10] was used to test the activity of E3 (A), E3.1B (B), E3.1BB (C) and E3.1A (D) enhancers. This plasmid enables the simultaneous detection of the electroporated cells (expressing mCherry) and cells specifically expressing the enhancer driven reporter (GFP). Ubiquitous expression of mcherry is demonstrated in cross sections of HH24 spinal cords, while expression of GFP in E3, E3.1B and E3.1BB is restricted to motor neurons and dorsal interneurons. E3.1A directs sporadic and non-specific expression of GFP (D). Scale Bar in C – 50 µm. (TIF) [file pone.0057960.s004.tif]
